# Early occurrence of influenza A epidemics coincided with changes in occurrence of other respiratory virus infections

**DOI:** 10.1111/irv.12348

**Published:** 2015-12-11

**Authors:** Liselotte van Asten, Paul Bijkerk, Ewout Fanoy, Annemarijn van Ginkel, Anita Suijkerbuijk, Wim van der Hoek, Adam Meijer, Harry Vennema

**Affiliations:** ^1^ Centre for Infectious Disease Control NetherlandsNational Institute for Public Health and the EnvironmentBilthovenThe Netherlands

**Keywords:** Influenza, respiratory syncytial virus, seasonality, time trends, viral interference, virus interaction

## Abstract

**Background:**

Viral interaction in which outbreaks of influenza and other common respiratory viruses might affect each other has been postulated by several short studies. Regarding longer time periods, influenza epidemics occasionally occur very early in the season, as during the 2009 pandemic. Whether early occurrence of influenza epidemics impacts outbreaks of other common seasonal viruses is not clear.

**Objectives:**

We investigated whether early occurrence of influenza outbreaks coincides with shifts in the occurrence of other common viruses, including both respiratory and non‐respiratory viruses.

**Methods:**

We investigated time trends of and the correlation between positive laboratory diagnoses of eight common viruses in the Netherlands over a 10‐year time period (2003–2012): influenza viruses types A and B, respiratory syncytial virus (RSV), rhinovirus, coronavirus, norovirus, enterovirus, and rotavirus. We compared trends in viruses between early and late influenza seasons.

**Results:**

Between 2003 and 2012, influenza B, RSV, and coronavirus showed shifts in their occurrence when influenza A epidemics occurred earlier than usual (before week 1). Although shifts were not always consistently of the same type, when influenza type A hit early, RSV outbreaks tended to be delayed, coronavirus outbreaks tended to be intensified, and influenza virus type B tended not to occur at all. Occurrence of rhinovirus, norovirus, rotavirus, and enterovirus did not change.

**Conclusion:**

When influenza A epidemics occured early, timing of the epidemics of several respiratory winter viruses usually occurring close in time to influenza A was affected, while trends in rhinoviruses (occurring in autumn) and trends in enteral viruses were not.

## Introduction

It has been suggested that annual epidemics of different viral infections can interfere with each other, but clear trends over long time periods and underlying mechanisms are not known.^1^, ^2^, ^3^, ^4^ A few population‐level studies in Europe were based on observations in one respiratory season only (the 2009 H1N1 pandemic) in which the annually recurring influenza epidemic occurred relatively early. With the occurrence of several early influenza A seasons in recent years, an exploration of longer time trends of different viruses was considered useful to gain more insight in the suggested relationship between circulating viruses. Understanding viral shifts and potential drivers thereof is relevant for further understanding whether certain viruses might promote or inhibit (pandemic) influenza spread and whether influenza vaccination could potentially affect trends in other respiratory viruses.

In Europe, influenza epidemics generally occur in winter, with the official start of the epidemic when influenza‐like illness (ILI) incidence in primary care sentinel surveillance exceeds an epidemic threshold (in combination with influenza A virus detection in clinical specimens collected from a subset of those ILI patients).^5^ In the Netherlands, the influenza epidemic threshold has been calculated at an ILI incidence of 5·1/10 000 for minimally two consecutive weeks which is usually not exceeded before the turn of the year,^5^, ^6^, ^7^, ^8^ but the timing of the first exceedance (i.e., start of the epidemic) can vary between November and March (based on data from 1970 to 2006).^9^ An extremely early influenza season was the 2009/2010 season when the influenza A(H1N1)pdm09 pandemic strain appeared and the ILI epidemic threshold was reached by early October (week 41). Such early occurrence may affect the circulation of other seasonal pathogens, and theories on possible interference between outbreaks of different respiratory viruses have been postulated to be a possible cause of delays in expected seasonal outbreaks of other respiratory viruses.^1^, ^2^, ^3^ While those earlier population‐level studies focused mainly on the possible interaction between influenza A virus and rhinovirus circulation, there may also be a relationship between influenza A and other prevalent viruses. Therefore, we investigated trends in several common viruses for which laboratory data were available from national surveillance in the Netherlands for a longer time period of up to 10 years including both respiratory and enteral viruses.

## Methods

### Laboratory data

We investigated trends in the reporting of common infectious respiratory and enteral viruses for which laboratory data were available for the 10‐year period from 2003 to 2012, or part thereof:

1. Influenza virus type A
2. Influenza virus type B
3. Respiratory syncytial virus (RSV)
4. Rhinovirus (2006 onwards)
5. Coronavirus (mid‐2005 onwards)
6. Norovirus (2007 onwards)
7. Enterovirus
8. Rotavirus

The data were available from the “Weekly Virological Records System” of the Dutch Working Group on Clinical Virology giving the number of positive laboratory diagnoses by year and week, but not providing data on the denominator nor on age or gender of the patient. Submitting laboratories are associated with either hospitals or regional laboratories to which both GPs and hospitals submit samples. The estimated proportion of all positive diagnostics captured by this national surveillance varies between the monitored pathogens and was estimated between 38% (for rotavirus) and 73% (for influenza virus) in a 2002 study of five pathogens.^10^ The types of tests used can differ between the submitting laboratories and over time. Respiratory viruses were detected in throat swabs mainly by PCR‐based methods in respiratory specimens from patients with respiratory disease symptoms. Enteric viruses were detected in fecal samples by EIA‐based methods (rotavirus, norovirus) and by PCR‐based methods (norovirus). Enterovirus was detected mainly by PCR and in the early years also by culturing, in throat swabs, CNF, and in fecal specimens collected from patients suspected for an enterovirus infection. Cross‐reaction between rhinovirus and enterovirus PCRs for throat swab samples cannot be excluded completely. When collected from patients suspected for enterovirus infection, typing of enteroviruses indicated that this was a minority. When collected from patients with respiratory symptoms, enteroviruses might be misidentified as rhinovirus, as occurred during enterovirus D68 outbreaks.^11^

### Selection of years with early and late influenza A epidemics

**Figure 1:**
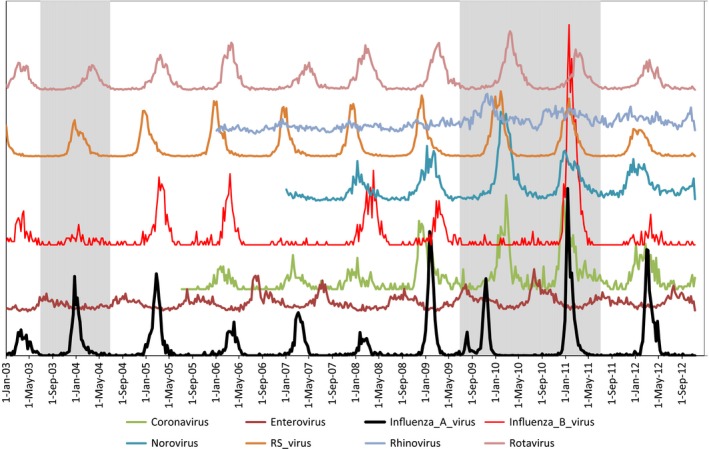
Time series of laboratory diagnoses* of common viruses 2003–2012 (early influenza A seasons depicted by gray‐shaded areas). *Absolute numbers of positive laboratory tests. The numbers of influenza A diagnoses were high during the 2009/2010 season due to the intensified testing that occurred during the A(H1N1)pdm09 pandemic and for graphical representation were reduced by a factor 13·1 in that season.

To determine whether an influenza A season occurred relatively early, we used both the influenza sentinel surveillance data and the laboratory data from the Weekly Virological Records system. As general practitioner (GP) influenza sentinel surveillance is the current gold standard for influenza surveillance in the Netherlands (with an epidemic threshold), early influenza A seasons were first identified using published dates of the influenza epidemics. We combined this information with visual inspection (as there is no threshold available) of trends in influenza A laboratory diagnoses reported in the Weekly Virological Records System for confirmation of the early ILI epidemics and for identification of any additional early influenza A seasons according to those laboratory data. A pre‐defined cutoff point for the identification of early influenza seasons is not available. Using the published ILI epidemic periods, we identified two relatively early influenza A seasons wherein influenza epidemics started before the turn of the year (i.e., before week 1): the 2003/2004 season (epidemic starting in week 49 lasting to week 4) and the 2009/2010 season (epidemic starting in week 41 lasting to week 51).^12^, ^13^ These two early epidemics were also confirmed by visible early increases in influenza laboratory data reported in the Weekly Virological Records System (Figure 1). After visual inspection of the laboratory data, the 2010/2011 season was additionally selected as an early season as influenza A diagnoses were clearly on the rise before week 1 (Figure 1). While the influenza epidemic in the 2008/2009 season did not start early (week 1)^8^ according to the ILI sentinel surveillance system, in the laboratory surveillance data this season seemed neither clearly early nor clearly late and was therefore excluded (influenza A laboratory diagnoses in this season are represented in Appendix 1).

### Presentation of virus trends

As most of the viruses under study peak in winter, we defined year‐long seasons running from July to June rather than using calendar years (January to December). For comparability between the seasons, we presented the number of detected viruses per week as a percentage of the total number of detections during the study period per each respective virus.

Due to the intensified testing that occurred during the A(H1N1)pdm09 pandemic, the total number of influenza A virus laboratory diagnoses in the pandemic season was 13·1 times higher than the average number of influenza A virus laboratory diagnoses in the 2003–2012 seasons, excluding the pandemic season. To facilitate the inclusion of such a high peak into the presented graphs, the number of influenza A virus laboratory diagnoses during the 2009/2010 season was scaled down (reduced by a factor 13·1, changing the height but not the shape of the epidemic in that year). As the number of influenza B virus laboratory diagnoses was very low during the pandemic, such downscaling was not performed for the influenza B virus laboratory diagnoses. Smoothing of time series was performed by calculating 3‐week moving averages (average of current, previous, and next week's value). Correlation coefficients between different viruses were calculated using Spearman's rank correlations for non‐normally distributed data. We also shifted the time series forwards and backwards in time (−10 to +10 weeks) and compared whether the time shift with maximal correlation differed between early and late influenza seasons.

## Results

### Virus seasonality and relative timing

The highest numbers of laboratory reports were available for RSV and the lowest for influenza B (20 282 and 1309 reported diagnoses during the total study period, Table 1). Time series of all considered pathogens are shown in Figure 1. Almost all of the included respiratory viruses (influenza A and B virus, RSV, coronavirus) except rhinovirus showed very clear seasonality in their reporting over time. The numbers increase, peak, and are elevated during winter season (December–February) or occasionally in very early spring (March–April) (Figure 1 and Appendix 2A–D). However, while influenza B virus diagnoses also displayed such winter seasonality, outbreaks did not occur each winter (five of the ten observed years showed clear influenza B epidemics, while the other 5 years showed very low levels or even near‐absence). In our data, rhinovirus levels showed less clear seasonality than the other respiratory viruses. They tended to be elevated over broad periods (Figure 1) and seemed to be present year‐round with less pronounced elevations during autumn/winter/spring. Generally, RSV epidemics preceded influenza A virus epidemics, which in turn preceded influenza B epidemics (in the years that influenza B epidemics occurred). Increases in coronavirus also usually preceded increases in influenza A (but not in the 2009/2010 season). The timing of rhinovirus levels relative to influenza A virus in laboratory diagnoses was less clear (due to seemingly year‐round presence of rhinoviruses in specimens submitted for laboratory diagnoses).

**Table 1:** Number of laboratory reports from the Weekly Virological Records system

Season^a^	Influenza A			Influenza B			RS virus			Coronavirus			Rhinovirus			Rotavirus			Norovirus			Enterovirus		
	Total	Mean	Max	Total	Mean	Max	Total	Mean	Max	Total	Mean	Max	Total	Mean	Max	Total	Mean	Max	Total	Mean	Max	Total	Mean	Max
2003/2004	538	10	92	52	1	6	1697	33	165	–	–	–	–	–	–	949	18	76	–	–	–	732	14	41
2004/2005	660	12	95	182	3	20	1980	37	208	–	–	–	–	–	–	1320	25	107	–	–	–	816	15	41
2005/2006	298	6	39	152	3	21	2249	43	252	84	2	8	306	12	22	1579	30	145	–	–	–	888	17	51
2006/2007	398	8	50	14	0	2	1959	38	225	90	2	12	750	14	35	1054	20	84	297	11	33	1454	28	82
2007/2008	206	4	27	199	4	22	2161	42	239	111	2	11	803	15	33	1829	35	137	1157	22	118	804	15	71
2008/2009	796	15	144	119	2	13	2465	47	278	279	5	24	1085	21	38	1862	36	144	2059	40	163	1066	21	52
2009/2010	514	10	89	22	0	3	3102	59	297	313	6	33	2253	43	94	2232	42	180	3617	68	265	1265	24	62
2010/2011	940	18	194	506	10	65	2732	53	264	402	8	31	2092	40	66	1507	29	124	2662	51	149	1538	30	97
2011/2012	785	15	123	58	1	9	1884	36	125	246	5	16	1810	35	64	1241	24	92	2536	49	124	1013	19	44
Total	5142			1309			20 282			1556			9646			13 654			12 919			10 191		

a Season: year‐long time periods running from July to June.

The two gastrointestinal viruses in the study (norovirus and rotavirus) both also presented with winter seasonality, often with norovirus preceding rotavirus epidemics (Figure 1 and Appendix 2C). Norovirus and influenza A virus epidemics usually overlapped, but the norovirus epidemics had broader peaks, and numbers tended to start increasing earlier than influenza A virus diagnoses levels and decreased after the disappearance of influenza A virus. Also, rotavirus outbreaks usually occurred before influenza A outbreaks started.

Enteroviruses, of which there are many types, can cause respiratory illness, but they also cause other illness.^14^ Enterovirus data were sparse until 2005, but in the 2006–2012 time period, enterovirus levels generally peak in summer (June–August).

### Description of viruses during early occurrence of influenza A virus

**Figure 2:**
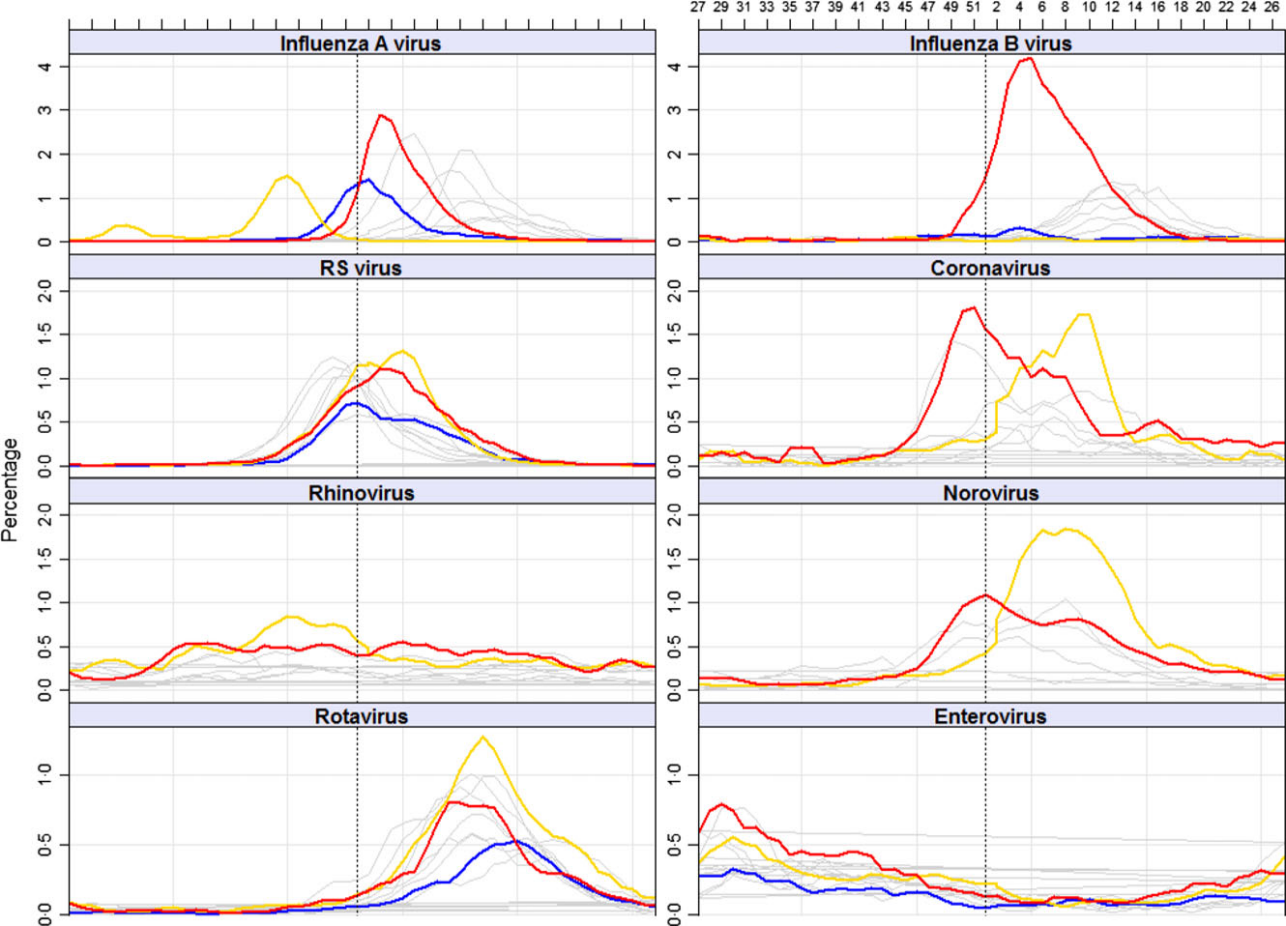
Three‐week moving averages of laboratory submissions for eight viruses stacked by season (2003/2004–2011/2012). Percentages are calculated over the total number of submissions per respective pathogen over the total study period. Influenza A counts during the 2009/2010 pandemic were scaled down to allow their fit into the graphs (see Methods). 2003/2004 data not available for coronavirus, norovirus, rotavirus, and enterovirus.

Visually, we assessed whether seasons with early influenza A virus epidemics (as reported in the Weekly Virological Records System) coincided with shifts in the reporting of other common pathogens or shifts in temperature and humidity trends. Occurrence of seasons with relatively early influenza A virus circulation compared to seasons with later circulation of influenza A virus is shown in Figure 2 (early seasons in color, late seasons in gray). For the other viruses, the same grouping was made (based on early and late influenza seasons), with their trend during early influenza A seasons also given in color (Figure 2).

#### Viruses with shifted trends: RSV, influenza B virus, coronavirus

Influenza B, RSV, and coronavirus (Figure 2) showed a shift when influenza A virus epidemics occurred early, but the shifts were not always of the same type (shifted in time, or in intensity) within each virus. Less clear differences in timing of peaks were observed for rhinovirus, norovirus, rotavirus, and enterovirus (Table 2 and Figure 2).

**Table 2:** Timing of highest peak of positive laboratory reports per virus (by yearly seasons running from July to June in 2003–2012)

	Absolute numbers		Smoothed data^a^	
	Influenza A season^b^		Influenza A season^b^	
	Early	Late	Early	Late
Influenza A virus	wk 46–wk 2	wk 4–wk 14	wk 46–wk 2	wk 5–wk 13
Influenza B virus^c^	wk 3	wk 10–wk 14	wk 3–wk 4	wk 10–wk 15
RS virus	wk 52–wk 4	wk 51–wk 52	wk 52–wk 4	wk 50–wk 52
Rhinovirus^d^	–	–	–	–
Coronavirus^e^	wk 51–wk 8	wk 1–wk 8	wk 51–wk 9	wk 1–wk 9
Norovirus e	wk 52–wk 4	wk 44–wk 51	wk 52–wk 7	wk 50–wk 3
Rotavirus	wk 8–wk 13	wk 8–wk 18	wk 8–wk 14	wk 9–wk 17
Enterovirus	wk 29–wk 31	wk 28–wk 38	wk 29–wk 30	wk 27–wk 37

a Three‐week moving average.

b Based on influenza‐like illness sentinel surveillance.

c Excluding two seasons due to virtual absence of B outbreaks (2006/2007, and 2009/2010).

d Most years do not show clear peaks for rhinovirus.

e Coronavirus data since 2005, norovirus data since 2007.

#### Influenza B virus: tendency of absence or earlier occurrence at higher levels

During early influenza A seasons, circulation of influenza B virus (i) was virtually absent in two of the three early influenza A seasons (2003/2004 and 2009/2010) or (ii) had a 6–7 weeks earlier occurrence and with much more intensive circulation than usual in 2010/2011. During the five late influenza A seasons, influenza B circulation failed to occur only once (2006/2007) (Figure 2).

#### RSV: tendency of delayed occurrence or decreased peak level

During two of the three early influenza A seasons, RSV activity occurred later than usual (Figure 2: 2009/2010 and 2010/2011 seasons, shifted 2–4 weeks [Table 2]). In 2003/2004, RSV timing was not unusual but peak level was lower than usual (almost half as low but slightly more spread out). During the five late influenza A seasons, RSV epidemics had such lower peak level only once (2006/2007) (Figure 2).

#### Coronavirus: tendency of higher levels

Data were available for two of the three early influenza seasons, and these two seasons coincide with increased coronavirus activity (of which the 2010/2011 season is shifted forward in time) (Figure 2).

#### Viruses showing no clear shifts: rhinovirus, norovirus, rotavirus, enterovirus

We observed no shifts in the seasonal trends of rotavirus and enterovirus (Figure 2). Rhinovirus trends did not change except for a slightly higher number of diagnoses in the 2009/2010 season which might be due to the intensified testing of patients with respiratory symptoms during the influenza A(H1N1)pdm09 pandemic season. Norovirus trends showed nothing exceptional except for a higher‐than‐usual peak number of diagnoses in the 2009/2010 season, which was a heavy norovirus season due to a new variant strain (Figure 2).

### Correlation between influenza A and other viruses at different time shifts

The correlation coefficients between influenza and other viruses were stratified by timing of the occurrence of the influenza epidemic (early versus late, Table 3). As the different viruses might differ in the timing of their occurrence relative to influenza A occurrence, the correlation coefficients were calculated at different time lags (shifted between 10 weeks earlier up to 10 weeks later) to determine the time shift with the maximum correlation with influenza A. When comparing seasons with early versus later occurrence of influenza epidemics, the maximum correlation coefficient was observed at different lags for all viruses. This difference in time lag relative to influenza A occurrence was smallest for influenza B, indicating that influenza B circulation relative to influenza A tends to occur at a more stable delay than for the other viruses. This suggests that changes in timing of influenza A circulation will be accompanied by changes in timing of influenza B circulation. However, such an overall correlation coefficient (calculated over multiple seasons combined) obscured the visual observation that in two of the three early influenza seasons, influenza B virus was almost absent (Figure 2).

**Table 3:** Correlation coefficients between number of weekly laboratory diagnoses of influenza A and weekly counts of other pathogens at different time lags (10 weeks later to 10 weeks earlier)^a^

No. of weeks pathogen is shifted with respect to influenza A counts	Influenza B		RSV		Coronavirus		Rhinovirus		Rotavirus		Norovirus		Enterovirus	
	Influenza season		Influenza season		Influenza season		Influenza season		Influenza season		Influenza season		Influenza season	
	Early	Late	Early	Late	Early	Late	Early	Late	Early	Late	Early	Late	Early	Late
10 weeks later	0·297	0·034	–0·223	0·229	0·013	**0**·**259**	–0·033	0·162	0·460	**0**·**426**	–0·094	0·290	–0·239	–0·277
9 weeks later	0·352	0·107	–0·151	0·299	0·064	0·232	–0·015	0·235	0·526	0·416	–0·056	**0**·**296**	–0·315	–0·300
8 weeks later	0·406	0·142	–0·068	0·364	0·086	0·242	–0·011	0·284	0·581	0·402	0·044	0·253	–0·382	–0·292
7 weeks later	0·422	0·174	0·006	0·417	0·169	0·225	0·043	0·349	0·642	0·374	0·119	0·215	–0·439	–0·282
6 weeks later	0·488	0·273	0·088	0·439	0·220	0·225	0·039	0·381	0·686	0·367	0·182	0·206	–0·517	–0·307
5 weeks later	0·549	0·273	0·177	0·491	0·337	0·188	0·058	0·415	0·709	0·344	0·234	0·165	–0·562	–0·276
4 weeks later	0·528	0·390	0·246	0·482	0·380	0·148	0·044	0·415	0·723	0·324	0·308	0·152	–0·596	–**0**·**312**
3 weeks later	**0**·**550**	0·377	0·318	0·519	0·430	0·177	0·069	0·498	0·741	0·276	0·348	0·126	–0·621	–0·310
2 weeks later	0·533	0·405	0·397	0·545	0·471	0·152	0·084	0·486	**0**·**747**	0·218	0·399	0·111	–**0**·**659**	–0·310
1 week later	0·511	**0**·**447**	0·464	**0**·**567**	0·519	0·192	0·091	0·502	0·737	0·179	0·443	0·098	–0·637	–0·279
Same week	0·527	0·445	0·520	0·542	0·561	0·140	0·138	**0**·**527**	0·724	0·127	0·476	0·100	–0·611	–0·293
1 week earlier	0·515	0·429	0·585	0·541	0·625	0·117	0·131	0·469	0·674	0·075	0·510	0·044	–0·632	–0·244
2 weeks earlier	0·481	0·440	0·621	0·506	0·615	0·161	0·156	0·495	0·630	0·021	0·530	0·014	–0·606	–0·186
3 weeks earlier	0·448	0·398	0·678	0·460	0·667	0·082	0·165	0·454	0·595	–0·050	0·527	0·010	–0·583	–0·157
4 weeks earlier	0·357	0·364	0·696	0·445	**0**·**675**	0·080	0·186	0·472	0·533	–0·123	0·519	–0·029	–0·531	–0·145
5 weeks earlier	0·345	0·347	0·740	0·362	0·670	0·083	0·213	0·437	0·453	–0·187	**0**·**532**	–0·041	–0·473	–0·124
6 weeks earlier	0·297	0·325	**0**·**766**	0·322	0·668	0·044	0·269	0·413	0·394	–0·253	0·508	–0·091	–0·411	–0·078
7 weeks earlier	0·248	0·263	0·757	0·305	0·629	–0·009	0·273	0·397	0·306	–0·317	0·505	–0·132	–0·359	–0·042
8 weeks earlier	0·190	0·265	0·749	0·240	0·614	–0·038	0·306	0·346	0·234	–0·370	0·448	–0·171	–0·299	–0·012
9 weeks earlier	0·151	0·195	0·733	0·188	0·534	–0·134	0·337	0·332	0·141	–0·444	0·407	–0·205	–0·209	0·067
10 weeks earlier	0·081	0·140	0·715	0·149	0·473	–0·169	**0**·**354**	0·286	0·052	–0·482	0·385	–0·252	–0·155	0·116

a Bolded numbers indicate the largest correlation coefficient (with a negative correlation for enterovirus due to opposite seasonality with influenza A virus).

## Discussion

Viruses that showed a shifted trend of reporting during years with early influenza A epidemics were of respiratory nature with clear winter seasonality and with epidemics occurring relatively close in time to influenza A virus epidemics. Although per individual virus the shifts were not consistently of the same type or direction, generally said, in years when influenza A hit early, RSV tended to be delayed, coronavirus outbreaks tended to be intensified, and influenza B virus tended not to circulate at all. Viruses that act on other organ systems (enteral) seemed not to be affected by the timing of influenza A outbreaks. In our data, rhinovirus showed little seasonality, but rather year‐round presence, and therefore, no association with timing of influenza A could be observed.

Changes in seasonal patterns of respiratory pathogen laboratory reports have been observed in other, shorter, studies. In Hong Kong, after the early occurring A(H1N1)pdm09 pandemic, increases were observed in adenovirus and parainfluenza virus detections, while the usual RSV summer peak disappeared in data from hospitals and clinics.^15^ Rhinovirus and coronavirus were not included. In another study in Beijing, all respiratory virus epidemics, except rhinovirus, were delayed after the pandemic influenza epidemic (data came from fever clinics which screen patients prior to being assigned to a specific hospital department).^16^ However, both regions have different climates from the Netherlands (and from each other) complicating comparisons. Other European observations have also been reported regarding the effect of rhinovirus on influenza virus circulation. While those reports hypothesize on viral interference between influenza virus and rhinovirus circulation (with rhinovirus delaying influenza spread),^1^, ^2^, ^3^, ^17^ we (like the Beijing study^16^) did not observe clear‐cut trends in rhinovirus reports in our laboratory diagnoses data. The relationship previously reported between rhinovirus and influenza virus might perhaps be age specific because in one study, the reduced likelihood of A(H1N1)pdm09 detection in rhinovirus‐positive samples^3^ was observed in a pediatric population, as was the reduced likelihood of detecting eight viruses in rhinovirus‐positive samples in another study in children,^17^ while our data were not age specific and we did not have such information on concurrent infections.

Besides the reported possible interaction between rhinovirus and influenza A virus, much less is known about interaction between and with other (respiratory and non‐respiratory) viruses. Our data suggest that respiratory viruses may impact each other's seasonality (in this study focused on the relationship between influenza A virus and other respiratory viruses), albeit through unknown mechanisms, as also hinted at by the Beijing and Hong Kong data.^15^, ^16^ Virtually all research suggesting interaction between respiratory viruses in human populations has focused on observational (ecological) studies such as ours, most including only one or a few seasons of data while our study included a 10‐year period. Only one prospective cohort study has been published (based on one season) which showed rhinovirus and coronavirus to interrupt the A(H1N1)pdm09 pandemic.^18^ Our results could not confirm their finding of coronavirus inhibiting influenza A virus circulation, but we observed more coronavirus laboratory reports when influenza A virus circulated early. In contrast to most of the other studies, we could investigate whether patterns recurred due to our longer study period. This revealed that coronavirus laboratory reporting was more intense in 2009/2010 when it did not overlap with influenza A virus circulation. However, it was also more intense in the early influenza A year thereafter (2010/2011) when it actually completely overlapped with the influenza A epidemic hinting that direct biological inhibition of either virus by the other might be unlikely and thus apparent interaction reported by others may have depended on indirect factors or may be spurious. These variations by year in our data illustrate how results from shorter observational studies have to be interpreted with caution, especially when they focus only on 1 year or one season. However, also in our longer study we have to remain cautious; to refute biological inhibition between coronavirus and influenza, it would be helpful to know whether the coronavirus and influenza virus specimens were from the same age group.

Unfortunately, as in other observational studies, surveillance artifacts could not be ruled out for any of the viruses in our study. These artifacts concern increases and decreases in laboratory testing over time that may not necessarily be related to changes in virus circulation in the population. Like previous studies, we did not have information on issues potentially underlying such changes, such as shifts in laboratory testing policy, availability and use of new diagnostic tools, variations in testing intensity due to previous or new outbreaks, and possible outsourcing of certain tests. Furthermore, information on age was not available in our data, possibly diluting trends that might have been visible were data investigated age specifically, or alternatively creating trends and shifts that might otherwise have been absent in certain age groups. If certain age groups are more likely to be hospitalized (such as infants with RSV infection) and/or to be sampled than other age groups and thus overrepresented in the laboratory data, our observed trends might not be representative for the total population if virus circulation trends actually differ between age groups. Future ecological analyses should preferably include age‐specific data as this may further clarify the true occurrence of shifted seasonality. Comparison between the different studies (including ours) is also made difficult by unknowns other than age, such as illness severity and influenza vaccination status.

As other ecological‐type studies have suggested, also our observed shifts in the occurrence of influenza B virus, RSV, and coronavirus during early influenza A seasons suggest that viral interaction might play a role in the occurrence of virus epidemics.^1^, ^2^, ^3^, ^17^ With our data, the precise direction of potential interactions is not clear, that is, which virus(es) impacts which. There are several types of virus–virus interactions, described in a review by Da Palma,^19^ ranging from (i) direct virus–virus interactions (e.g., when nucleic acids or proteins of one virus physically interact with the genes or gene products of a coinfecting virus); (ii) indirect interactions resulting from alterations in the host environment; to (iii) immunological interactions. However, with the current data we cannot determine whether the viruses impacted each other biologically. Further, there are other issues that can impact the timing and magnitude of seasonal epidemics such as environmental, social, and behavioral phenomena that may also be the potential drivers of observed shifts in reporting patterns instead of viral interaction (alone).^20^ To further attempt to disentangle these issues, future ecological studies should preferably include age‐specific virological data over multiple seasons and better yet, cohort studies could be performed that (i) address the order of infection by different viruses (by serial sampling of subjects during the study period regardless of symptoms) as proposed by others;^17^, ^21^ (ii) include persons from the same household as was done by Pascalis *et al*.;^18^ and (iii) include multiple years and thus multiple seasons per virus.

There are several examples highlighting the importance of understanding drivers of viral shifts. As suggested by Greer *et al*., further studies are relevant in light of the discussion whether rhinovirus promotes (pandemic) influenza spread through coughing and sneezing^22^ or whether it may paradoxically provide natural protection due to inhibition of influenza infection in those already infected with rhinovirus.^17^ Further studies may also provide input to the question whether influenza vaccination can render persons more susceptible to other respiratory viruses due to the lack of temporary non‐specific immunity induced by actual influenza infection.^23^ Canadian and Australian researchers hypothesize that through this same mechanism of induced temporary non‐specific immunity, the circulation of a seasonal influenza strain preceding a pandemic strain might decrease the susceptibility to that ensuing pandemic strain.^24^ Not evaluated in our study but possibly also playing a role in virus shifts or virus–virus interactions is the variation in the circulation of influenza A subtypes from year to year. Evaluation of such an effect might require evaluation of longer time series as the first general impression from our data was that there was no clear consistent association with subtype as the three early influenza years were not all dominated by the same influenza A type (H3N2 in 2003/2004, H1N1pdm09 in 2009/2010, and H1N1pdm09 in 2010/2011).^13^, ^25^ Further, drift variants of influenza A virus were present both in early and in late influenza A seasons^25^ and would require closer scrutiny (such as the timing and proportion of drift variant occurrence within seasons and if possible ensuing disease severity) to determine their possible role in disrupted respiratory virus trends.

Studying the association of climatic factors (an example of environmental phenomena potentially affecting virus circulation) with influenza^26^, ^27^, ^28^ was beyond the scope of this study, although covering the timespan of the study, a visual assessment of temperature and humidity trends showed no clear visual association between early influenza occurrence and early occurrence of low temperatures or low humidity. Although two of the three early ILI seasons (2009/2010, 2010/2011) seem to have longer and colder cold spells (average weekly temperatures below 0°C) that also occur earlier in the winter season than in the other years, the first mentioned season (the 2009/2010 pandemic year) actually showed the cold spell occurring *after* the influenza season instead of at the beginning or during the influenza season (Appendix 2E).

Unlike earlier studies, we included several non‐respiratory pathogens, for which the results showed no shifts in trends, and therefore, interaction with influenza A virus seems unlikely. As norovirus, rotavirus, and enterovirus affect other organ systems, direct interaction may not be expected. However, sporadic examples do not rule out such unexpected interactions as others have suggested that live polio vaccine (against an enteral virus) might prevent otitis media^29^ (a respiratory infection), and decrease infantile diarrhea mortality^30^ (gastro‐intestinal mortality). Another issue is that, like others, we investigated shifts in virus circulation within the same seasons. Whether early occurrence of influenza A may affect the circulation of seasonal pathogens in seasons thereafter is not known.

In conclusion, when influenza hit early we observed shifts in patterns of several respiratory pathogens that occur close in time with influenza A. Further research is needed to understand whether this was caused by (in)direct biological interaction between viruses or other underlying mechanisms such as human behavior and environmental factors or whether these observed shifts were just random occurrences. Understanding these phenomena is of value in understanding or predicting the timing and magnitude of viral epidemics, providing knowledge which can be used for early warning and the allocation of healthcare resources.

## Figures and Tables

**Figure A1:**
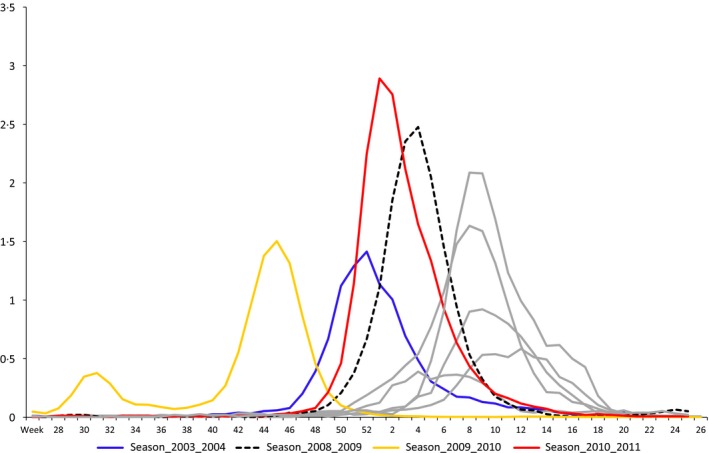
Three‐week moving average of influenza A diagnoses by season (2003/2004—2011/2012).

**Figure A2:**
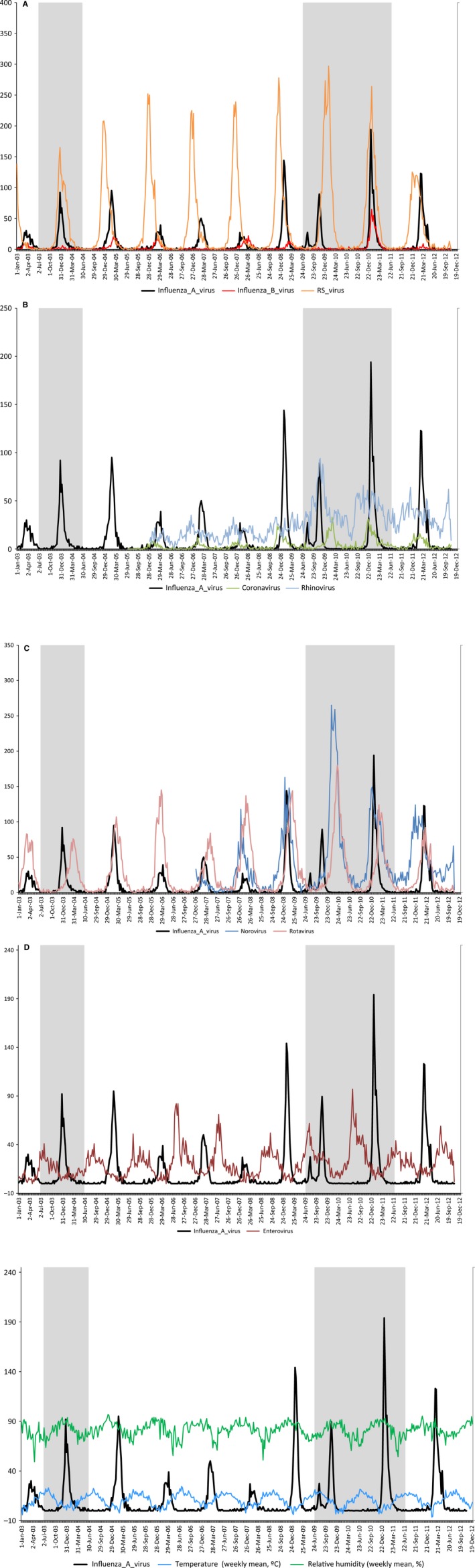
(A) Time series of influenza A and B and RS virus (early influenza A seasons depicted by gray‐shaded areas). (B) Time series of influenza A, rhinovirus, and coronavirus (early influenza A seasons depicted by gray‐shaded areas). (C) Time series of influenza A, norovirus, and rotavirus (early influenza A seasons depicted by gray‐shaded areas). (D) Time series of influenza A and enterovirus (early influenza A seasons depicted by gray‐shaded areas). (E) Time series of influenza A, mean temperature, and mean relative humidity (early influenza A seasons depicted by gray‐shaded areas).

## References

[1] Linde A , Rotzen‐Ostlund M , Zweygberg‐Wirgart B , Rubinova S , Brytting M . Does viral interference affect spread of influenza? Euro Surveill 2009; 14:19354. 19822124

[2] Anestad G , Nordbo SA . Interference between outbreaks of respiratory viruses. Euro Surveill 2009; 14:19359. 19883536

[3] Casalegno JS , Ottmann M , Duchamp MB *et al* Rhinoviruses delayed the circulation of the pandemic influenza A (H1N1) 2009 virus in France. Clin Microbiol Infect 2010; 16:326–329. 20121829 10.1111/j.1469-0691.2010.03167.x

[4] Casalegno JS , Ottmann M , Bouscambert‐Duchamp M , Valette M , Morfin F , Lina B . Impact of the 2009 influenza A(H1N1) pandemic wave on the pattern of hibernal respiratory virus epidemics, France, 2009. Euro Surveill 2010; 15:19485. 20158981

[5] Vega T , Lozano JE , Meerhoff T *et al* Influenza surveillance in Europe: establishing epidemic thresholds by the moving epidemic method. Influenza Other Respir Viruses 2013; 7:546–558. 22897919 10.1111/j.1750-2659.2012.00422.x PMC5855152

[6] de Jong JC , Rimmelzwaan GF , Donker GA , Meijer A , Fouchier RA , Osterhaus AD . [The 2006/'07 influenza season in the Netherlands and the vaccine composition for the 2007/'08 season]. Ned Tijdschr Geneeskd 2007; 151:2158–2165. 17957994

[7] Rimmelzwaan GF , de Jong JC , Donker GA , Meijer A , Fouchier RA , Osterhaus AD . [Influenza season 2007/'08 in the Netherlands: antigenic variation, oseltamivir resistance and vaccine composition for the 2008/'09 season]. Ned Tijdschr Geneeskd 2008; 152:2138–2144. 18856032

[8] Donker GA . Continue Morbiditeits Registratie Peilstations Nederland 2008. 2009.

[9] Donker G , Gravestein J . De beste tijd voor griepvaccinatie. Huisarts en Wetenschap 2007; 50:2.

[10] van den Brandhof WE , Kroes ACM , Bosman A , Peeters MF , Heijnen MLA . [Reporting virus diagnostics in the Netherlands: representativeness of the virological weekly reports]. Infectieziekten Bull 2002; 13:110–113.

[11] Jaramillo‐Gutierrez G , Benschop KS , Claas EC *et al* September through October 2010 multi‐centre study in the Netherlands examining laboratory ability to detect enterovirus 68, an emerging respiratory pathogen. J Virol Methods 2013; 190:53–62. 23458694 10.1016/j.jviromet.2013.02.010

[12] Rimmelzwaan GF , de Jong JC , Bartelds AI , Wilbrink B , Fouchier RA , Osterhaus AD . [The 2003/2004 influenza season in the Netherlands with a limited epidemic of the virus variant A/Fujian, and the vaccine composition for the 2004/2005 season]. Ned Tijdschr Geneeskd 2004; 148:1984–1988. 15524136

[13] de Jong JC , Rimmelzwaan GF , Donker GA , Meijer A , van der Hoek W , Osterhaus ADME . De Mexicaanse grieppandemie van 2009: een overzicht met focus op Nederland. Nederlands Tijdschrift voor Medische Microbiologie 2011; 19:6–11.

[14] Abzug MJ . The enteroviruses: problems in need of treatments. J Infect 2014; 68(Suppl 1):S108–S114. 24119825 10.1016/j.jinf.2013.09.020

[15] Mak GC , Wong AH , Ho WY , Lim W . The impact of pandemic influenza A (H1N1) 2009 on the circulation of respiratory viruses 2009–2011. Influenza Other Respir Viruses 2012; 6:e6–e10. 22212717 10.1111/j.1750-2659.2011.00323.x PMC5657134

[16] Yang Y , Wang Z , Ren L *et al* Influenza A/H1N1 2009 pandemic and respiratory virus infections, Beijing, 2009‐2010. PLoS One 2012; 7:e45807. 23029253 10.1371/journal.pone.0045807 PMC3447804

[17] Greer RM , McErlean P , Arden KE *et al* Do rhinoviruses reduce the probability of viral co‐detection during acute respiratory tract infections? J Clin Virol 2009; 45:10–15. 19376742 10.1016/j.jcv.2009.03.008 PMC7185458

[18] Pascalis H , Temmam S , Turpin M *et al* Intense co‐circulation of non‐influenza respiratory viruses during the first wave of pandemic influenza pH1N1/2009: a cohort study in Reunion Island. PLoS One 2012; 7:e44755. 22984554 10.1371/journal.pone.0044755 PMC3440351

[19] DaPalma T , Doonan BP , Trager NM , Kasman LM . A systematic approach to virus‐virus interactions. Virus Res 2010; 149:1–9. 20093154 10.1016/j.virusres.2010.01.002 PMC7172858

[20] Fisman D . Seasonality of viral infections: mechanisms and unknowns. Clin Microbiol Infect 2012; 18:946–954. 22817528 10.1111/j.1469-0691.2012.03968.x

[21] Rhedin S , Hamrin J , Naucler P *et al* Respiratory viruses in hospitalized children with influenza‐like illness during the H1n1 2009 pandemic in Sweden. PLoS One 2012; 7:e51491. 23272110 10.1371/journal.pone.0051491 PMC3522717

[22] Merler S , Poletti P , Ajelli M , Caprile B , Manfredi P . Coinfection can trigger multiple pandemic waves. J Theor Biol 2008; 254:499–507. 18606170 10.1016/j.jtbi.2008.06.004 PMC7094108

[23] Cowling BJ , Fang VJ , Nishiura H *et al* Increased risk of noninfluenza respiratory virus infections associated with receipt of inactivated influenza vaccine. Clin Infect Dis 2012; 54:1778–1783. 22423139 10.1093/cid/cis307 PMC3404712

[24] Kelly H , Barry S , Laurie K , Mercer G . Seasonal influenza vaccination and the risk of infection with pandemic influenza: a possible illustration of non‐specific temporary immunity following infection. Euro Surveill 2010; 15:19722. 21144441 10.2807/ese.15.47.19722-en

[25] Meijer A , Rimmelzwaan GF , Dijkstra F , Donker GA . [Recent developments with regard to influenza; flu‐watchers in action] Actuele ontwikkelingen betreffende influenza; griepspotters in actie. Tijdschrift voor Infectieziekten 2009; 4:9.

[26] te Beest DE , van Boven M , Hooiveld M , van den Dool C , Wallinga J . Driving factors of influenza transmission in the Netherlands. Am J Epidemiol 2013; 178:1469–1477. 24029683 10.1093/aje/kwt132

[27] Sooryanarain H , Elankumaran S . Environmental role in influenza virus outbreaks. Annu Rev Anim Biosci 2015; 3:347–373. 25422855 10.1146/annurev-animal-022114-111017

[28] Skog L , Linde A , Palmgren H , Hauska H , Elgh F . Spatiotemporal characteristics of pandemic influenza. BMC Infect Dis 2014; 14:378. 25011543 10.1186/1471-2334-14-378 PMC4226939

[29] Seppala E , Viskari H , Hoppu S *et al* Viral interference induced by live attenuated virus vaccine (OPV) can prevent otitis media. Vaccine 2011; 29:8615–8618. 21939720 10.1016/j.vaccine.2011.09.015 PMC7127548

[30] Contreras G . Effect of the administration of oral poliovirus vaccine on infantile diarrhoea mortality. Vaccine 1989; 7:211–212. 2781855 10.1016/0264-410x(89)90230-2

